# Inferring FDG-PET-positivity of lymph node metastases in proven lung cancer from contrast-enhanced CT using radiomics and machine learning

**DOI:** 10.1186/s41747-022-00296-8

**Published:** 2022-09-15

**Authors:** Boris Gorodetski, Philipp Hendrik Becker, Alexander Daniel Jacques Baur, Alexander Hartenstein, Julian Manuel Michael Rogasch, Christian Furth, Holger Amthauer, Bernd Hamm, Marcus Makowski, Tobias Penzkofer

**Affiliations:** 1 Department of Radiology (including Pediatric Radiology), Charité–Universitätsmedizin Berlin, corporate member of Freie Universität Berlin and Humboldt-Universität zu Berlin, Campus Virchow-Klinikum, Augustenburger Platz 1, 13353 Berlin, Germany; 2 Department of Nuclear Medicine, Charité–Universitätsmedizin Berlin, corporate member of Freie Universität Berlin and Humboldt-Universität zu Berlin, Campus Virchow-Klinikum, Augustenburger Platz 1, 13353 Berlin, Germany; 3 Berlin Institute of Health (BIH), Anna-Louisa-Karsch-Str. 2, 10178 Berlin, Germany

**Keywords:** Machine learning, Lymph nodes, Lymphatic metastasis, Lung neoplasms, Tomography (x-ray computed)

## Abstract

**Background:**

We evaluated the role of radiomics applied to contrast-enhanced computed tomography (CT) in the detection of lymph node (LN) metastases in patients with known lung cancer compared to ^18^F-fluorodeoxyglucose positron emission tomography (PET)/CT as a reference.

**Methods:**

This retrospective analysis included 381 patients with 1,799 lymph nodes (450 malignant, 1,349 negative). The data set was divided into a training and validation set. A radiomics analysis with 4 filters and 6 algorithms resulting in 24 different radiomics signatures and a bootstrap algorithm (Bagging) with 30 bootstrap iterations was performed. A decision curve analysis was applied to generate a net benefit to compare the radiomics signature to two expert radiologists as one-by-one and as a prescreening tool in combination with the respective radiologist and only the radiologists.

**Results:**

All 24 modeling methods showed good and reliable discrimination for malignant/benign LNs (area under the curve 0.75−0.87). The decision curve analysis showed a net benefit for the least absolute shrinkage and selection operator (LASSO) classifier for the entire probability range and outperformed the expert radiologists except for the high probability range. Using the radiomics signature as a prescreening tool for the radiologists did not improve net benefit.

**Conclusions:**

Radiomics showed good discrimination power irrespective of the modeling technique in detecting LN metastases in patients with known lung cancer. The LASSO classifier was a suitable diagnostic tool and even outperformed the expert radiologists, except for high probabilities. Radiomics failed to improve clinical benefit as a prescreening tool.

**Supplementary Information:**

The online version contains supplementary material available at 10.1186/s41747-022-00296-8.

## Key points

- Radiomics applied to contrast-enhanced computed tomography is feasible in detecting lymph node metastases in patients with proven lung cancer.
- The least absolute shrinkage and selection operator (LASSO) classifier is suitable as a diagnostic tool applied to radiomics in this setting.
- Radiomics failed to improve clinical benefit as a prescreening tool.

## Background

Lung cancer is the most common cause of cancer-related death in the world with an incidence of 2.2 million and cancer-related death of 1.9 million people in 2017 [1]. The vast majority have non-small cell lung cancer (NSCLC), specifically, adenocarcinoma [2, 3] and are diagnosed either with advanced local or metastatic disease with a limited 5-year survival rate of 8−18% and above 50% in the case of localised disease [4–6].

Precise tumour staging is not only important for outcome classification, but it is crucial for choosing the best therapeutic regime between resection, radiotherapy, chemotherapy, and/or immunotherapy for the respective patient [7–9]. Therefore, multiple non-invasive staging modalities such as computed tomography (CT) and/or positron emission tomography (PET) as well as invasive staging modalities such as mediastinoscopy and/or endobronchial ultrasound transbronchial needle aspiration are used for classifying tumour extension. In addition, tumour biomarkers such as genomic analysis (*e.g.*, epidermal growth factor receptor mutation, anaplastic lymphoma kinase gene fusion) or protein expression (*e.g.*, programed cell death ligand 1) play an important role for tailoring treatment for an individual patient [10–12].

Even though PET/CT is a cost-effective imaging modality in the primary staging of NSCLC [13] PET/CT and invasive staging modalities are still rather expensive. Therefore, restaging is often only performed via CT, especially in regions where PET/CTs are not broadly available, and there is a considerable need for alternative non-invasive diagnostic tools for precise personalised medicine. In order to fill this diagnostic gap, radiomics, an approach translating biological tissue characterisation into quantitative image analyses, has been developed. With the assistance of radiomics medical diagnosis are transferred from subjective qualitative assessment into more reliable and generalised objective quantitative assessment [14–18]. The results in disease detection, diagnosis, evaluation of prognosis and prediction of treatment response are encouraging and sometimes even overcome prediction of routine clinical detection tools [16, 18–22].

Currently, there is great interest in using radiomics for improving clinical decisions in lung cancer [16]. Most radiomics studies on lung cancer have been focused on the primary tumour, *e.g.*, for differentiating between benign and malignant lung lesions [23, 24] or between primary and secondary tumours [25], survival prediction [26–28], treatment response [29, 30], or predicting lymph node (LN) metastases on the morphology of the primary tumour [7]. There have been some radiomics analysis on LN metastases for other tumour entities such as gastric cancer [31], head and neck cancer [32], or bladder cancer [16]. However, to the best knowledge of the authors, only some minor metric analysis with a comparison of CT-based density measured in Hounsfield units, short and long axis diameter and three-dimensional volumetry [33] has been performed in lung cancer.

Therefore, our study sought to evaluate the potential diagnostic role of radiomics in the detection of LN metastases in patients with lung cancer with contrast-enhanced (CE) CT. [^18^F]Fluorodeoxyglucose (FDG)-PET served as a reference. Primary endpoint was defined as predicting LN metastases, secondary endpoint to assess the clinical benefit of radiomics with a decision curve analysis in comparison to expert radiologists, and tertiary endpoint to assess the potential of radiomics as a prescreening tool for expert radiologists, whether it improves clinical decision from the radiologists if radiomics preselects the lymph nodes.

## Methods

### Study cohort

This retrospective single-institution study was conducted in compliance with the Health Insurance Portability and Accountability Act and approved by the institutional review board. Between December 2011 and May 2018, a total of 733 patients with histologically proven primary lung cancer–adenocarcinoma (*n* = 440, 60%) or squamous-cell carcinoma (*n* = 293, 40%) received FDG-PET/CT within 100 days after date of diagnosis. Only patients with adequate (*i.e.*, contrast enhanced, good imaging quality) CE-CT performed as part of FDG-PET/CT were included in the final cohort. Out of these 381 patients, 228 patients (60%) had adenocarcinoma and 153 patients (40%) had squamous-cell carcinoma. A total of 1,799 LNs (450 PET-positive, 1,349 PET-negative) was included in the analysis (Table 1).

**Table 1:** Demographics

Parameter		Number (%)
Total population		381 (100.0)
Sex	Male	238 (53.1)
	Female	143 (46.9)
Age	< 60 years	102 (26.8)
	≥ 60 years	279 (73.2)
NSCLC	Adenocarcinoma	228 (60.0)
	Squamous-cell carcinoma	153 (40.0)
Stage	IA	75 (19.8)
	IB	47 (12.4)
	IIA	27 (7.1)
	IIB	27 (7.1)
	IIIA	70 (18.5)
	IIIB	39 (10.3)
	IIIC	2 (0.5)
	IVA	79 (20.8)
	IVB	13 (3.4)
Lymph node analysis		1,799 (100%)
Lymph nodes	PET-positive	450 (43.0)
	PET-negative	1,349 (18.2)
Thoracic lymph node station	1	98 (5.4)
	2	250 (13.9)
	3	177 (9.8)
	4	761 (42.3)
	5	207 (11.5)
	6	142 (7.9)
	7	110 (6.1)
	8	10 (0.6)
	9	0 (0.0)
	10	44 (2.4)

*NSCLC* Non-small cell lung cancer, *PET* Positron emission tomography

### Imaging technique

FDG-PET/CT imaging was performed with a dedicated PET/CT scanner (Gemini TF 16; Philips, Amsterdam, The Netherlands) with time-of-flight capability (Philips Astonish TF technology). Patients had to fast for ≥ 6 h prior to the injection of [^18^F]FDG, and a blood glucose level ≤ 190 mg/dL was ensured. A mean of 310.17 MBq [^18^F]FDG ± 51.06 MBq (± standard deviation) was injected intravenously. The PET scan was performed after an uptake time of 91.72 ± 28.03 min (mean ± standard deviation). PET data was acquired from base of skull to the proximal femora in three-dimensional acquisition mode (emission, 90 to 180 s per bed position; bed overlap 53.3%). Attenuation correction and anatomical mapping were either based on unenhanced low-dose CT (automated tube current modulation; maximum tube current-time product 50 mAs; tube voltage 120 kVp; gantry rotation time 0.5 s) or contrast-enhanced diagnostic CT (automated tube current modulation; maximum tube current-time product 200 mAs; tube voltage 120 kVp; delay after contrast agent injection 80 s; bolus injection rate 3 mL/s).

PET raw data were reconstructed using iterative reconstruction (ordered subset expectation maximisation) with time-of-flight analysis (BLOB-OS-TF; iterations 3; subsets 33; filter ‘smooth’; matrix 144 × 144; voxel size 4.0 × 4.0 × 4.0 mm^3^). CT raw data were reconstructed with a soft tissue convolution kernel and a slice thickness of 5 mm for attenuation correction or 3 mm for visual assessment and radiomics analysis, respectively.

### Imaging data evaluation

The Medical Imaging Interaction Toolkit (MITK v. 2016.11, DKFZ, Heidelberg, Germany) (34) was used to randomly select and to semiautomatically segment LNs from CE-CT. Only mediastinal and hilar lymph nodes were included. Two experienced radiologists in the field of hybrid imaging (with more than 5 years of experience, respectively) classified these LNs as either malignant (PET-positive) or benign (PET-negative) according to the maximum standardised uptake value (SUVmax) corrected for total body mass on FDG-PET (Supplementary Figure S1). Readers used the threshold SUVmax > 2.5 to classify LNs, which is the most common threshold used for LNs in NSCLC [9]. In an initial step, LNs with SUVmax < 2 were uniformly considered PET-negative, and LNs with SUVmax > 3 were considered PET-positive. In a second step, all remaining lymph nodes with SUVmax between 2 and 3 were thoroughly investigated for potential errors in SUVmax calculation due to misalignment between PET and CT or due to interference of activity from adjacent tissue (*e.g.,* oesophagus or vessel walls). If such interference could be excluded, the LN was rated as positive or negative based on SUVmax > 2.5. If the SUVmax was potentially erroneous, the reader classified the lymph node based on visual assessment of the PET images. During visual assessment, LNs were usually considered positive if uptake was above mediastinal background, especially if the mediastinal pattern of FDG-avid LNs was asymmetrical and in the drainage channel of the primary tumour localisation. This additional step was introduced to minimise the number of potentially misclassified LNs. For the test cohort, the selected lymph nodes were evaluated based on the CE-CT by two expert radiologists in the field of diagnostic oncology (with more than 5 years of experience, respectively) using the size (> 10 mm in short axis according to RECIST v1.1) [34] and/or the configuration (texture, border, and shape) [35] and classified as benign, likely benign, likely malignant, or malignant. This reading step was performed blinded to the PET-results and to avoid recall bias performed > 3 months after the initial read.

### Statistical analysis

**Fig. 1:**
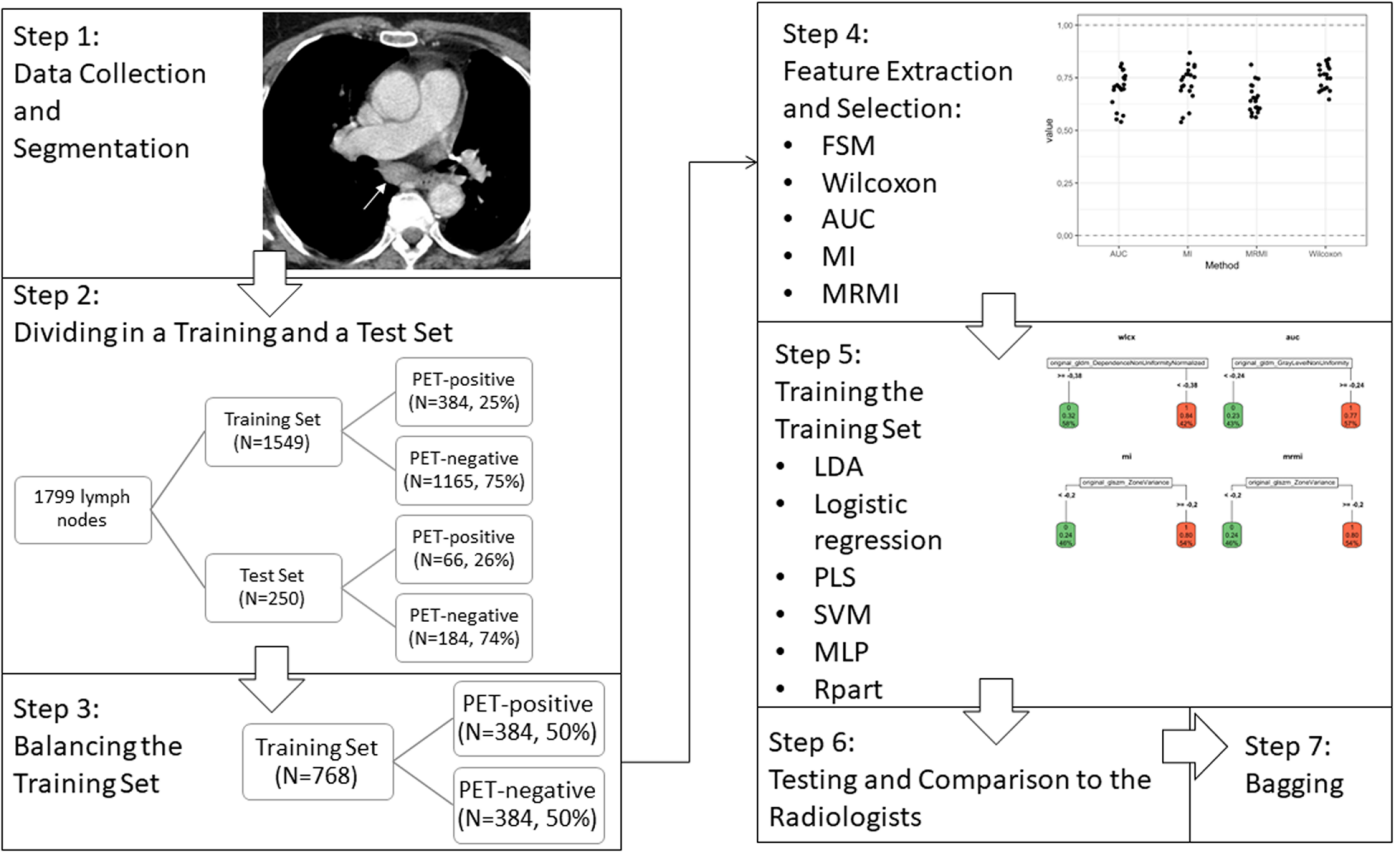
Flowchart: balancing, feature selection, bagging. *AUC* Area under the curve, *FSM* Feature selection method, *LDA* Linear discriminant analysis, *MI* Mutual information, *MLP* Multilayer perceptron (neuronal network), *MRMI* Maximum relevance minimum redundancy, *PET* Positron emission tomography, *PLS* Partial least squares, *Rpart* Recursive partition, *SVM* Support vector machine

The data set was divided into a training (384, 25%, PET-positive and 1,165, 75%, PET-negative LNs) and an internal validation test set (66, 26%, PET-positive and 184, 74%, PET-negative LNs) and balanced in order to prevent both overfitting and underfitting. For comparison reason the features according to the Image Biomarker Standardization Initiative, IBSI [36, 37] were extracted using the filters feature selection method (FSM), Wilcoxon, area under the curve (AUC), mutual information, and maximum relevance minimum redundancy by PyRadiomics [38]. The extracted features were standardised, reduced to 20 features and tested for stability for each filter separately. Training was performed with the training set using six algorithms: linear discriminant analysis (LDA); logistic regression; partial least squares (PLS), support vector machine [SVM], neuronal network (multilayer perceptron); and recursive partition. Each filter resulted into 24 different modeling methods for quantitative analysis. For internal validation, all 24 modelling methods were tested in the internal validation test set and compared to the clinical assessment of both expert radiologists. Additionally, it was examined whether radiomics improved the performance of the radiologist in the uncertain group (likely benign and likely malignant). To encounter for potential bias through randomly balancing, the training and test sets were aggregated using a bootstrap algorithm (bagging) with 30 bootstrap iterations (Fig. 1).

**Fig. 2:**
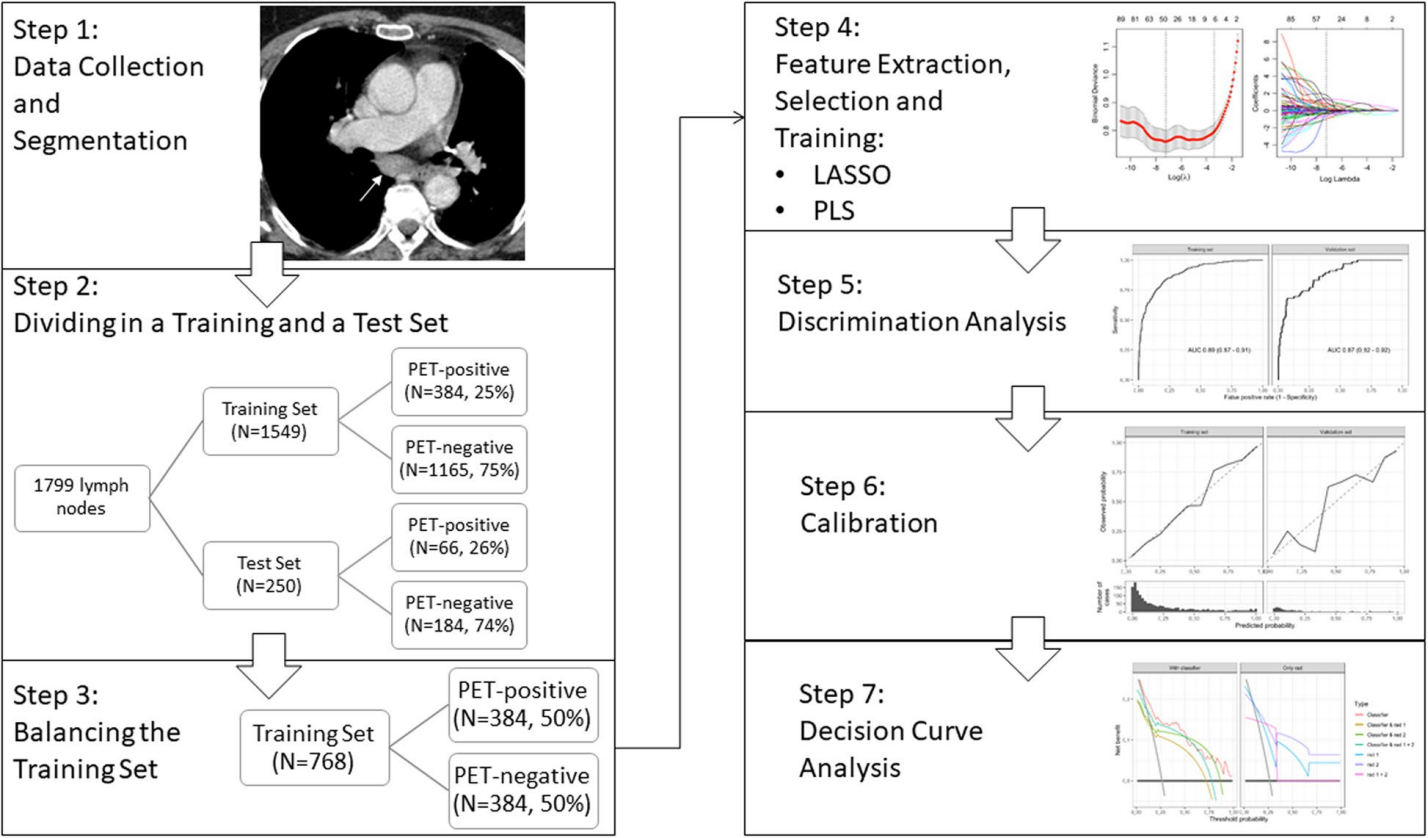
Flowchart: in-depth analysis, clinical utility, and decision curve analysis. *LASSO* Least absolute shrinkage and selection operator, *PET* Positron emission tomography, *PLS* Partial least squares

**Fig. 3:**
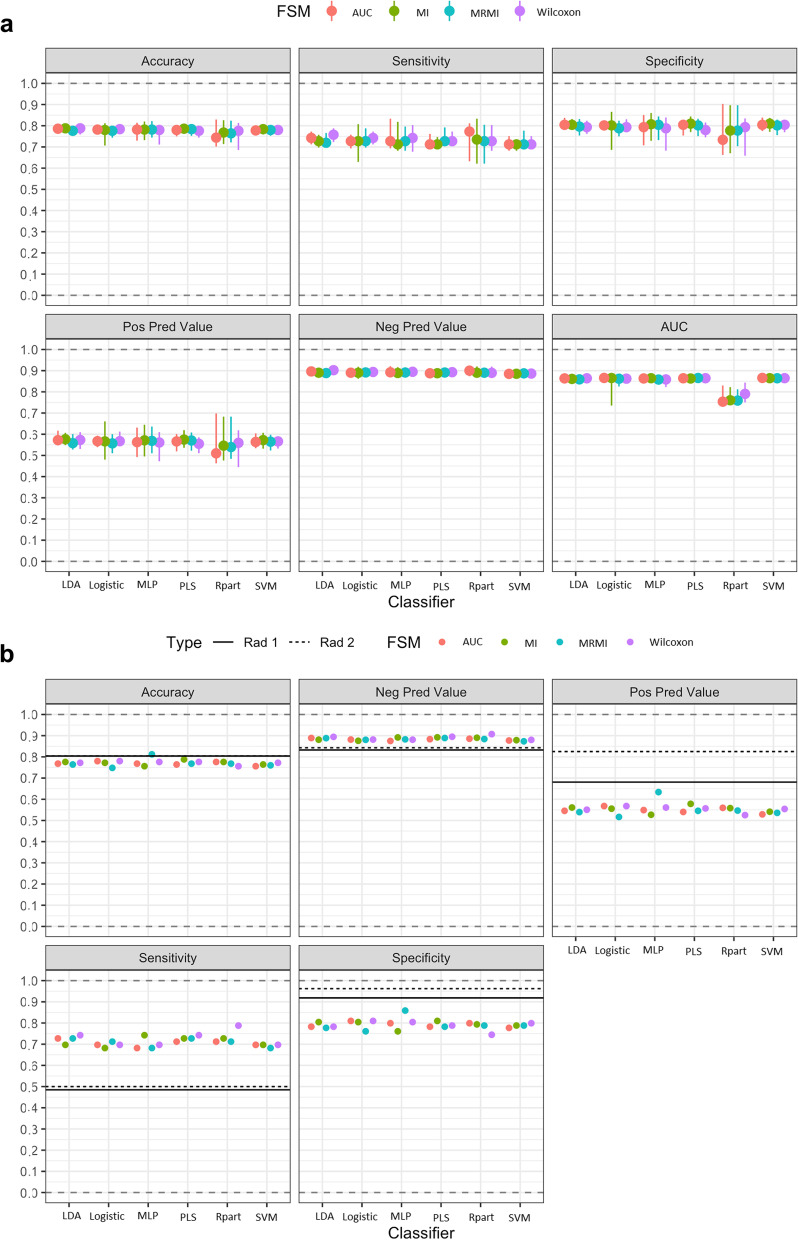
Performance of radiomics after bagging with 30 times of bootstrap iterations (**a**), in comparison to expert radiologists (**b**). *AUC* Area under the curve, *FSM* Feature selection method, *LDA* Linear discriminant analysis, *Logistic* Logistic regression, *MI* Mutual information, *MLP* Multilayer perceptron (neuronal network), *MRMI* Maximum relevance minimum redundancy, *NPV* Negative predictive value, *PLS* Partial least squares, *PPV* Positive predictive value, *Rpart* Recursive partition, *SVM* Support vector machine

In order to evaluate the clinical impact of radiomics we performed an in-depth analysis using PLS as a classifier that has shown to be reliable with good accuracy and AUC in the balanced and bootstrap iteration method (Table 2, Fig. 2, Fig. 3a) and the least absolute shrinkage and selection operator (LASSO; Supplementary Figure S2) logistic regression algorithm that has shown good results in similar studies with other tumour entities [7, 16].

**Table 2:** Performance comparison of radiomics after bagging with 30 times of bootstrap iterations

Classifier	FSM	Accuracy	Sensitivity	Specificity	PPV	NPV	AUC
LDA	AUC	0.79 (0.77−0.81)	0.74 (0.71−0.77)	0.8 (0.78−0.84)	0.57 (0.55−0.62)	0.9 (0.89−0.91)	0.86 (0.85−0.87)
LDA	MI	0.79 (0.77−0.8)	0.73 (0.7−0.76)	0.8 (0.79−0.83)	0.58 (0.55−0.61)	0.89 (0.88−0.9)	0.86 (0.85−0.87)
LDA	MRMI	0.78 (0.76−0.8)	0.72 (0.7−0.77)	0.8 (0.75−0.83)	0.56 (0.53−0.6)	0.89 (0.88−0.9)	0.86 (0.85−0.87)
LDA	Wilcoxon	0.79 (0.76−0.81)	0.76 (0.72−0.79)	0.79 (0.76−0.82)	0.57 (0.53−0.61)	0.9 (0.89−0.91)	0.86 (0.86−0.87)
Logistic regression	AUC	0.78 (0.76−0.8)	0.73 (0.69−0.76)	0.8 (0.78−0.82)	0.57 (0.54−0.59)	0.89 (0.88−0.9)	0.87 (0.85−0.87)
Logistic regression	MI	0.78 (0.71−0.81)	0.73 (0.63−0.81)	0.8 (0.69−0.87)	0.57 (0.48−0.66)	0.89 (0.86−0.92)	0.87 (0.74−0.88)
Logistic regression	MRMI	0.78 (0.74−0.8)	0.73 (0.7−0.79)	0.79 (0.75−0.82)	0.56 (0.51−0.6)	0.89 (0.88−0.91)	0.86 (0.83−0.87)
Logistic regression	Wilcoxon	0.78 (0.77−0.81)	0.74 (0.71−0.77)	0.79 (0.78−0.83)	0.57 (0.54−0.61)	0.89 (0.89−0.91)	0.86 (0.85−0.87)
MLP	AUC	0.78 (0.73−0.81)	0.73 (0.69−0.83)	0.79 (0.71−0.85)	0.56 (0.49−0.63)	0.89 (0.88−0.92)	0.86 (0.85−0.87)
MLP	MI	0.78 (0.73−0.82)	0.71 (0.68−0.82)	0.81 (0.73−0.86)	0.57 (0.5−0.64)	0.89 (0.87−0.92)	0.86 (0.85−0.87)
MLP	MRMI	0.78 (0.74−0.82)	0.73 (0.68−0.8)	0.8 (0.73−0.84)	0.57 (0.51−0.64)	0.89 (0.87−0.91)	0.86 (0.84−0.87)
MLP	Wilcoxon	0.78 (0.71−0.8)	0.74 (0.68−0.8)	0.79 (0.68−0.84)	0.56 (0.47−0.61)	0.89 (0.88−0.91)	0.86 (0.82−0.88)
PLS	AUC	0.78 (0.75−0.8)	0.71 (0.7−0.76)	0.8 (0.75−0.83)	0.57 (0.52−0.6)	0.89 (0.88−0.9)	0.86 (0.85−0.87)
PLS	MI	0.79 (0.76−0.81)	0.71 (0.7−0.75)	0.81 (0.77−0.84)	0.57 (0.54−0.62)	0.89 (0.88−0.9)	0.86 (0.85−0.87)
PLS	MRMI	0.78 (0.75−0.8)	0.73 (0.71−0.79)	0.8 (0.75−0.83)	0.57 (0.52−0.61)	0.89 (0.88−0.91)	0.87 (0.86−0.87)
PLS	Wilcoxon	0.78 (0.74−0.79)	0.73 (0.71−0.77)	0.78 (0.75−0.82)	0.55 (0.51−0.59)	0.89 (0.88−0.91)	0.86 (0.86−0.87)
Rpart	AUC	0.74 (0.7−0.83)	0.77 (0.63−0.81)	0.73 (0.66−0.9)	0.51 (0.46−0.7)	0.9 (0.87−0.92)	0.75 (0.75−0.83)
Rpart	MI	0.77 (0.71−0.83)	0.73 (0.62−0.83)	0.78 (0.67−0.9)	0.55 (0.48−0.68)	0.89 (0.87−0.92)	0.76 (0.74−0.82)
Rpart	MRMI	0.76 (0.72−0.82)	0.73 (0.62−0.8)	0.78 (0.7−0.9)	0.54 (0.48−0.68)	0.89 (0.87−0.91)	0.76 (0.74−0.81)
Rpart	Wilcoxon	0.78 (0.69−0.81)	0.73 (0.68−0.8)	0.79 (0.66−0.83)	0.56 (0.44−0.62)	0.89 (0.88−0.92)	0.79 (0.75−0.84)
SVM	AUC	0.78 (0.76−0.8)	0.71 (0.68−0.75)	0.8 (0.78−0.84)	0.56 (0.53−0.6)	0.88 (0.88−0.9)	0.87 (0.86−0.87)
SVM	MI	0.78 (0.76−0.8)	0.71 (0.68−0.74)	0.81 (0.77−0.84)	0.57 (0.53−0.61)	0.89 (0.88−0.89)	0.86 (0.86−0.87)
SVM	MRMI	0.78 (0.75−0.8)	0.71 (0.7−0.78)	0.8 (0.76−0.83)	0.56 (0.52−0.6)	0.89 (0.88−0.91)	0.86 (0.85−0.88)
SVM	Wilcoxon	0.78 (0.76−0.79)	0.71 (0.69−0.75)	0.8 (0.77−0.82)	0.57 (0.53−0.59)	0.89 (0.88−0.9)	0.86 (0.86−0.87)

Data are given as median and 95% confidence interval. *AUC* Area under curve at receiver operating characteristics analysis, *FSM* Feature selection method, *LDA* Linear discriminant analysis, *MI* Mutual information, *MLP* Multilayer perceptron (neuronal network), *MRMI* Maximum relevance minimum redundancy, *NPV* Negative predictive value, *PLS* Partial least squares, *PPV* Positive predictive value, *Rpart* Recursive partition, *SVM* Support vector machine

For both classifiers we performed the corresponding feature selection and training method and generated a radiomics signature. For discrimination, a receiver operator characteristics curve was plotted and an AUC identified for the respective training and internal validation set. We performed a calibration analysis, calculated the Brier score [14] and performed a confusion matrix and metric analysis. To assess the clinical benefit, a decision curve analysis was plotted for the respective classifier, for the classifier as a prescreening tool in combination with the respective radiologist and only the radiologists (Fig. 2).

All statistical analyses were conducted in R 3.6.1 (R Core Team, 2019) on a x86_64-apple-darwin15.6.0 system under macOS Catalina 10.15.4 using the add-on packages knitr, readxl, tidyverse, mRMRe, caret, MASS, rpart, rpart.plot, pROC, kableExtra, pls, glmnet, rms, pROC, pathwork, and DescTools.

## Results

After balancing, feature selection, training in the training set and internal validation in the test set, reasonable prediction of malignant (PET-positive) LNs could be established irrespective of the 24 different modeling methods compared to the clinical assessment of the two expert radiologists with an accuracy of 0.75−0.81 and 0.80−0.80, a sensitivity of 0.68−0.79 and 0.48−0.50, a specificity of 0.74−0.86 and 0.92−0.96, respectively (Fig. 3b, Supplementary Table S1). Comparing radiomics to the clinical assessment of the two expert radiologists in the uncertain group (likely benign, likely malignant), radiomics has achieved significantly higher sensitivity but significantly lower specificity (Supplementary Figure S3, Supplementary Figure S4). Radiomics has not improved the clinical assessment of the two expert radiologists in the uncertain group (likely benign, likely malignant) with an accuracy of 0.77−0.83 and 0.78−0.82, a sensitivity of 0.68−0.73 and 0.65−0.71, and a specificity of 0.79−0.83 and 0.82−0.89, respectively (Supplementary Figure S3, Supplementary Table S2).

After bagging with 30 times of bootstrap iterations for different combinations of feature selection methods and classifiers resulting in 24 different modeling methods, a good and reliable discrimination between malignant and benign LNs was obtained, with an AUC of 0.75-0.87, a sensitivity of 0.71−0.77, a specificity of 0.73−0.80, a positive predictive value of 0.54−0.58, and a negative predictive value of 0.88−0.90. The smallest 95% confidence interval (CI) for AUC have been observed for the classifiers LDA and PLS (Table 2, Fig. 3a).

**Fig. 4:**
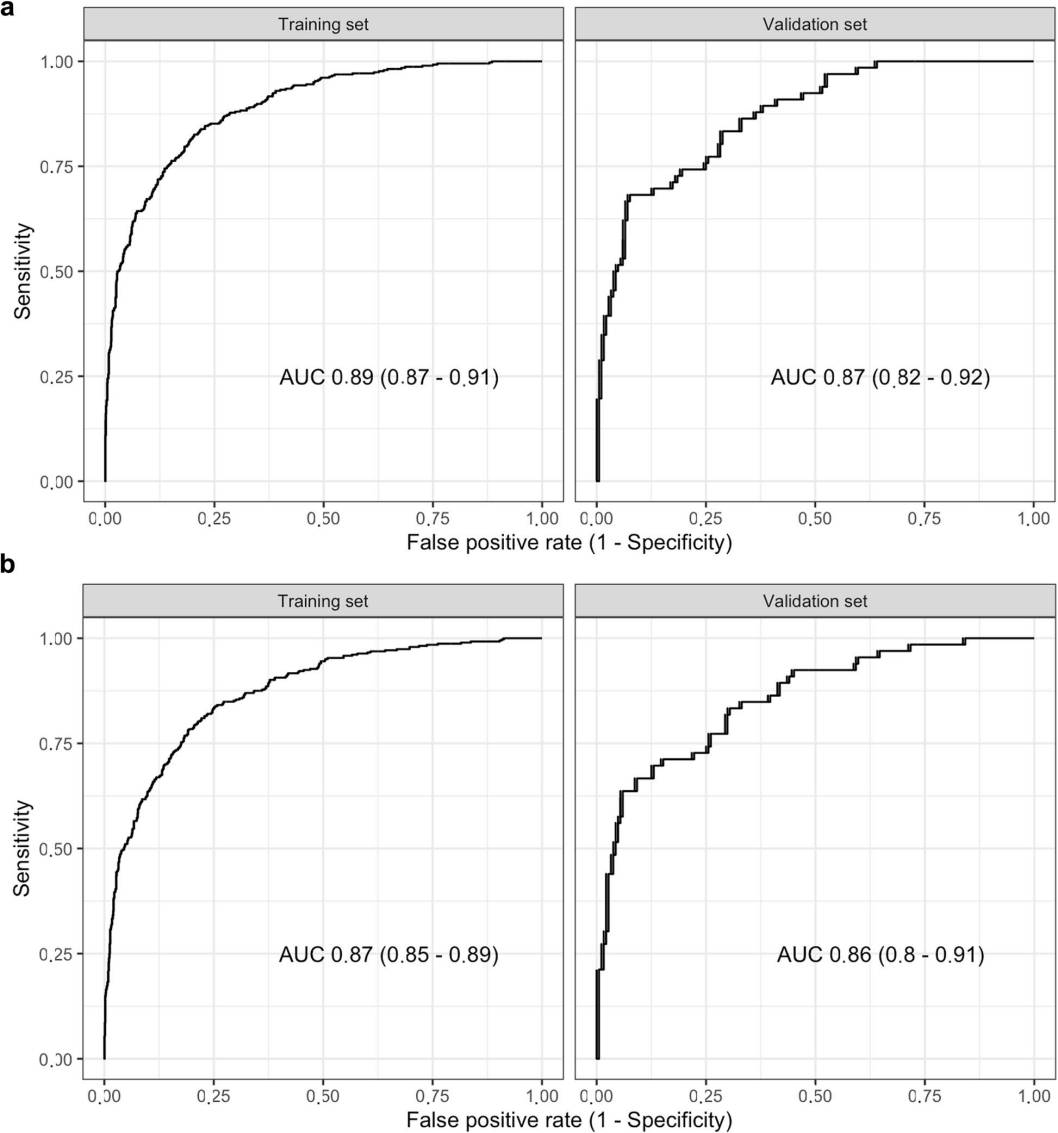
Discrimination analysis for the in-depth radiomics analysis. Receiver operating characteristics analysis after LASSO (**a**) and after PLS (**b**). *LASSO* Least absolute shrinkage and selection operator, *PLS* Partial least squares

**Fig. 5:**
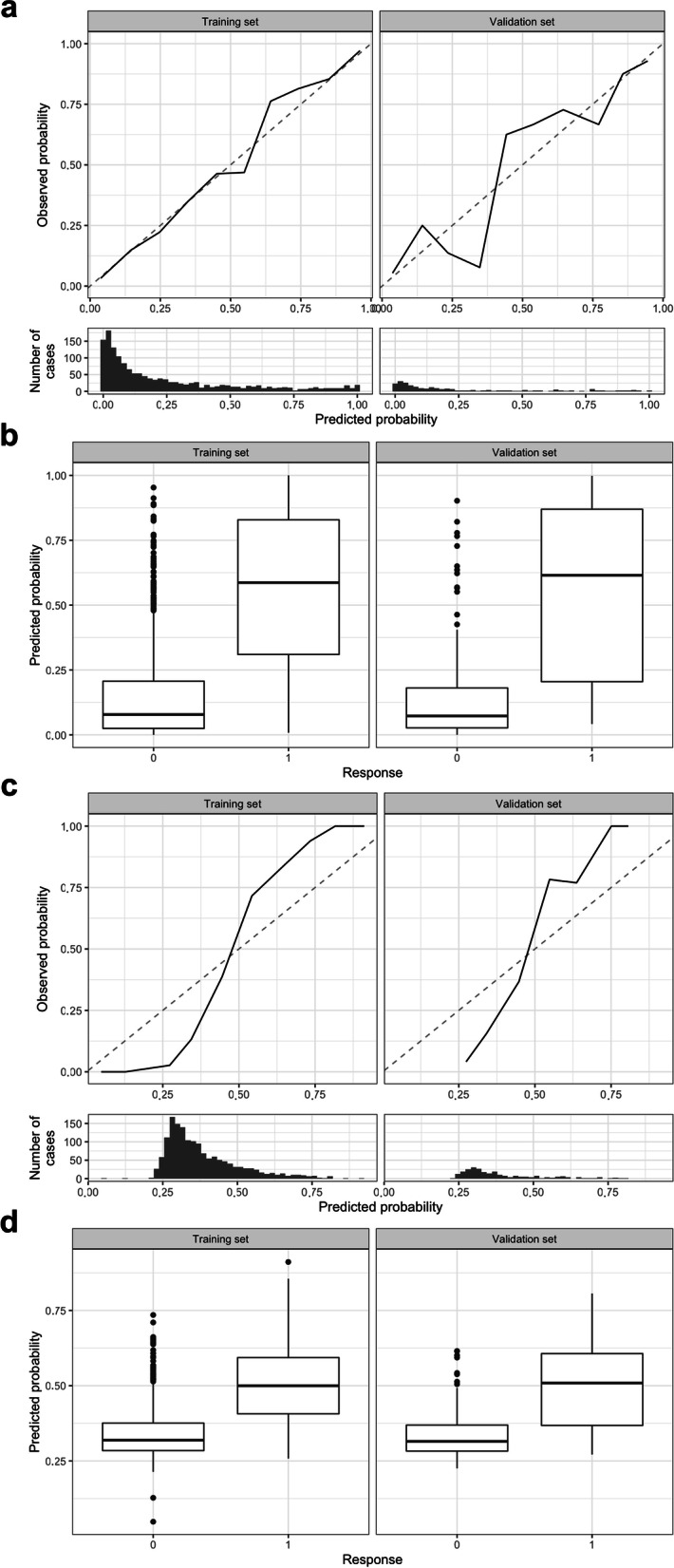
Calibration analysis for the in-depth radiomics analysis. Calibration plot after LASSO (**a**), box plot differentiating benign and malign lymph nodes after LASSO (**b**), calibration plot after PLS (**c**), and box plot differentiating benign and malign lymph nodes after PLS (**d**). *LASSO* Least absolute shrinkage and selection operator, *PLS* Partial least squares

In the in-depth analysis using LASSO and PLS for feature selection and training, good discrimination in the training (AUC 0.89, 95% CI 0.87−0.90 and 0.87, 95% CI 0.85−0.89, respectively) and validation set (AUC 0.87, 95% CI 0.82−0.92 and 0.86, 95% CI 0.80−0.91, respectively) has been achieved (Fig. 4). For LASSO calibration has been acceptable in the training set and having some deviation in the validation set. In contrast, PLS had some systematic deviation in the calibration plot and has not been using the entire probability range (Fig. 5). Similarly, the Brier score has been better for LASSO than for PLS in both the training (0.11 and 0.16) and validation set (0.12 and 0.16), respectively. After generating the best threshold for the predicted probabilities for LASSO and PLS, the confusion matrix and metric analysis have shown only a slightly higher accuracy (0.80 and 0.81) than the no information rate (0.736, *p* = 0.011 and 0.736, *p* = 0.005) in the validation set, respectively.

**Fig. 6:**
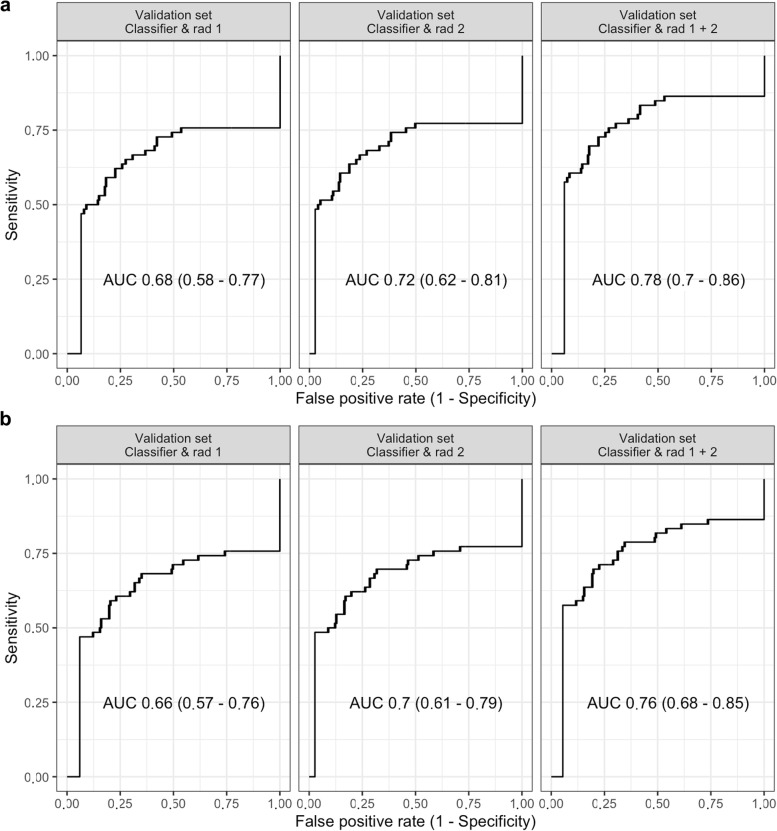
Discrimination analysis for the in-depth radiomics analysis using the radiomics signature as a prescreening tool for each of the two radiologists and the combination of both radiologists. Receiver operating characteristics analysis after LASSO (**a**) and after PLS (**b**). *LASSO* Least absolute shrinkage and selection operator, *PLS* Partial least squares

**Fig. 7:**
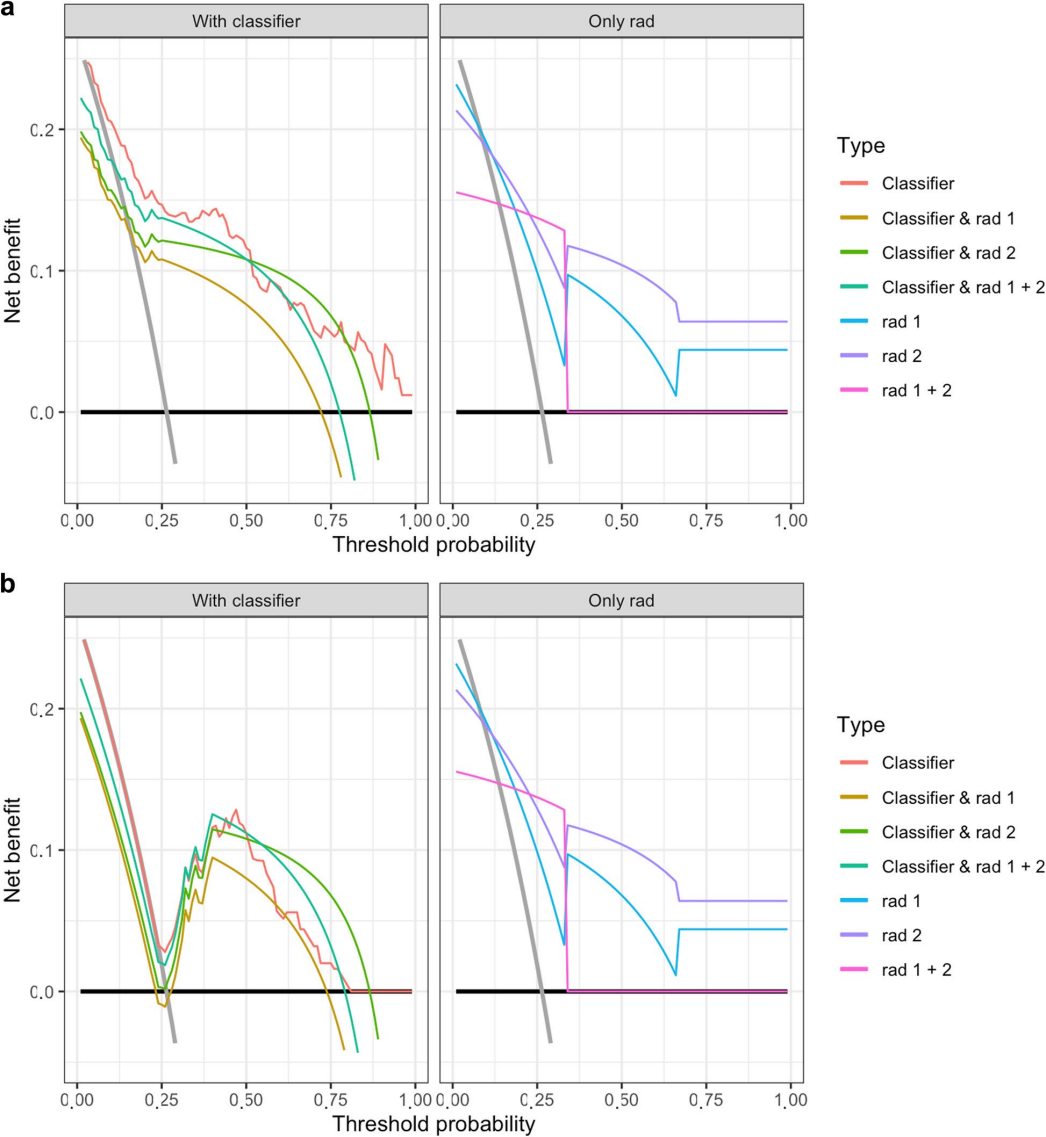
Assessment of the clinical benefit of radiomics as a prescreening tool for each of the two radiologists and the combination of both using a decision curve analysis. Decision curve analysis after LASSO (**a**) and after PLS (**b**). The grey line represents the strategy to classify all lymph nodes as malignant, the black line represents the strategy to classify all lymph nodes as benign. *LASSO* least absolute shrinkage and selection operator, *PLS* partial least squares

Using radiomics as a prescreening tool has reduced the discrimination with an AUC of 0.68−0.78 and 0.66-0.76 for the different combinations of the radiologists with lasso and pls (Fig. 6), respectively, and have not improved net benefit in the decision curve analysis (Fig. 7). Irrespective of the strategy to classify all LNs as either malignant or benign, the decision curve analysis clearly have shown a net benefit for LASSO in contrast to PLS or the radiologists for the entire probability range. Except for high probabilities, LASSO have shown a clear net benefit compared to the radiologists (Fig. 7).

## Discussion

Our study showed good results for radiomics applied to CE-CT in predicting LN metastases in patients with histopathologically proven lung cancer irrespective of the modeling method using traditional statistical metrics. A clear clinical benefit could only be asserted for LASSO in comparison to expert radiologists. Radiomics failed to make a clear benefit as a prescreening tool in this clinical scenario.

The discrimination power to differentiate benign (PET-negative) LNs and LN metastases (PET-positive) irrespective of the combination of classifier and feature selection method (*e.g.*, PLS with AUC 0.86, 95% CI 0.85−0.87 or LDA with Wilcoxon AUC 0.86, 95% CI 0.86−0.87) within our study is in line with other published studies with other tumour entities, and the narrow 95% CI in the internal validation set reflects a more robust assessment compared to studies in patients with gastric cancer (AUC 0.82, 95% CI 0.72−0.92) [31] or bladder cancer (AUC 0.85, 95% CI 0.72−0.99) [16].

Radiomics has already shown promising results with reasonable or good accuracy or discrimination for different steps in the complex universe of diagnostic work-up [23], outcome prediction [39] or therapy monitoring [4] in patients with lung cancer. It can reliably predict the development of lung cancer in a screening CT within 1 year with an accuracy of 80.1% (AUC 0.83) and within 2 years with an accuracy of 78.7% (AUC 0.72) [23]. Radiomics is even able to incorporate (semi-)automatically different resources such as clinical information and CT [16] or different modalities such as CT and PET and leads to a significant better discrimination of malignant and benign lung lesions comparing PET/CT (AUC 0.89, sensitivity 0.81, specificity 0.82) to CT (AUC 0.82, sensitivity 0.74, specificity 0.74; *p* = 0.018) [40]. However, though no significant difference in the performance between the PET radiomics signature (AUC 0.87) and the PET/CT (AUC 0.89) radiomics nomogram could be observed and the proof of the synergic clinical benefit is still pending, the performance solely of CT radiomics (AUC 0.82) is reasonable and might be an economical and timesaving alternative in the future [40]. Similarly, the clinical benefit of an individualised nomogram resulting from the combination of clinical information and the CT-based radiomics signature is still under discussion. Some improvement from the radiomics signature to the combined nomogram has been suggested, although not significant, in predicting LN metastases in bladder cancer (AUC 0.85 and 0.90) [16] or predicting LN metastases from the primary lesion in lung cancer (AUC 0.80, sensitivity 0.72, specificity 0.86 and AUC 0.86, sensitivity 0.92, specificity 0.82) [7] for the CE-CT-based radiomics signature and the radiomics nomogram incorporating the clinical information in the validation cohort, respectively.

In the current study, the accuracy of predicting LN metastases did not change if combining the radiomics signature and the clinical assessment of the two expert radiologists in equivocal LN (Supplementary Figure S3). More specifically, combined accuracy did not benefit from the significantly better sensitivity of the radiomics signature or the significantly better specificity of the expert radiologists (Supplementary Figure S3, and Figure S4). Nevertheless, the present study clearly shows that after bagging and the usage of different combination of classifiers and FSMs except for recursive partition, a good discrimination (AUC 0.86−0.87), and reasonable accuracy (0.78−0.79), sensitivity (0.71−0.74), and specificity (0.79−0.81) can be reached.

In contrast to other studies [3] in which radiomics (AUC 0.80, 95% CI 0.65−0.94; accuracy 0.71, sensitivity 0.74, and specificity 0.68) outperformed two expert radiologists (AUC 0.61 and 0.60) in differentiating benign and malignant lung lesions, the current study showed similar performance of radiomics and radiologists in the diagnosis of malignant and benign LNs using traditional statistical metrics such as AUC for discrimination, accuracy, sensitivity, and specificity. However, as these measurements are only theoretical, and the clinical benefit is affected by the calibration no direct information about the clinical value is given [41]. Therefore, direct inter-study comparison without a calibration analysis is difficult [41–43]. In the present study, the additional calibration plot, the Brier score and especially the confusion matrix depict this problem. Using LASSO as a classifier, a good discrimination (AUC 0.89 and 0.87 in the training and validation set, respectively) has been obtained, but the confusion matrix and statistical analysis in the validation set revealed only a slightly higher accuracy, even though significant, of 0.80 than the no information rate of 0.736 (*p* = 0.011), thus, clearly diminishing the benefit. The decision curve analysis in our in-depth analysis encounters this problem by incorporating the clinical consequences, the discrimination and calibration. It resulted into a net benefit (clinical benefit) comparing the respective analysis to the strategy to classify all LNs as either malignant or all as benign (Fig. 7) [41–43].

The classifier LASSO clearly showed a clinical benefit for all probabilities of PET-positive LNs and does no harm. Thus, this radiomics method is suitable as a diagnostic tool. It even outperforms the expert radiologist except for the high probability LN metastases. In contrast, PLS did not only perform worse than LASSO but even harms in the very low and high probability range as opposed to the estimation of the traditional statistical metrics as generally used by most radiomics analysis (AUC 0.87 and 0.86 in the training and validation set, respectively). Thus, within this study, using a decision curve analysis and generating a clinical benefit, PLS resulted not to be suitable as a diagnostic tool. Despite the net benefit of LASSO and the higher sensitivity throughout all radiomics signatures in contrast to the radiologists, the classifier LASSO failed to improve net benefit as a prescreening tool and even showed to harm under this constellation in the high probability range.

In a nutshell, the results of the present study contribute into another novel cornerstone in the diagnostic work-up for lung cancer with leaving the subjective interpretation and going one step further to objective and repeatable quantitative imaging using radiomics. The clear net benefit in the decision curve analysis of the classifier LASSO as a radiomics signature in differentiating between malignant and benign LNs might be not only crucial for classifying as stage N0, N1, or N2 at diagnosis in the future, but is also important for example to pave the way for a tumour biopsy for genomic analysis or detecting de novo LN metastases during follow-up.

There are some limitations to our study. First, we used the clinical standard FDG-PET/CT as a reference for classifying LNs as malignant and benign. According to the Cochrane meta-analysis [9], FDG-PET/CT is a very good screening tool with a summary sensitivity and specificity of 81.3% (95% CI 70.2−88.9%) and 79.4% (95% CI 70%−86.5%), respectively. However, it is still under the threshold of 95% comparing to the histopathological analysis. Second, average PET uptake time was above the recommended standard of 60 min. This might have resulted in increased LN SUVmax compared to an earlier imaging time point due to the irreversible nature of cellular FDG uptake. Comparability with other centers would therefore benefit from uniform uptake times. To encounter these methodological limitations, we included a huge number of analysed LNs (*n* = 1,799). Third, we have included only patients with histologically proven primary lung cancer, specifically adenocarcinoma or squamous-cell carcinoma, therefore, the results cannot be generalised on all primary lung cancer types. However, firstly the vast majority had NSCLC, specifically, adenocarcinoma [2, 3] and secondly, this is a preliminary study. Therefore, our promising results should encourage for further radiomics and machine learning analysis in future studies including other primary lung cancer types such as small cell lung cancer. Fourth, our analysis was based on a retrospective cohort. Therefore, selection bias and confounders cannot be fully excluded. To overcome them, we performed balancing and a multi-modeling methodology implementation with 30 times of bootstraps iterations and aggregation with 24 different combinations of classifiers and FSM. To encounter for further miscalibration and to assess the clinical benefit, we performed a decision curve analysis that incorporates both discrimination and calibration and is less sensitive to miscalibration. Depending on the model scenery, miscalibration reduces the net benefit (clinical utility); therefore, in a larger and better calibrated cohort, the clinical benefit of LASSO as a radiomics signature might be even higher than in the present study [42].

In conclusion, radiomics is feasible and showed good discrimination irrespective of the modeling technique in detecting LN metastases in patients with known lung cancer. The classifier LASSO showed to be suitable as a diagnostic tool and even outperforms the expert radiologists, except for high probabilities. Radiomics failed to improve clinical benefit as a prescreening tool.

## Supplementary Information

**Additional file 1: Supplementary Figure 1**. Representative lymph node metastases. **Supplementary Figure 2**. Training and cross-validation with LASSO. **Supplementary Figure 3**. Performance comparison of radiomics in the unsure group (likely benign and likely malignant) to expert radiologists and the effect of encountering the prediction model. **Supplementary Figure 4**. Performance comparison of radiomics in the unsure group (likely benign and likely malignant) to expert radiologists plotting the 95% confidence interval *AUC*. **Supplementary Table 1**. Performance comparison of radiomics and the two expert radiologists. **Supplementary Table 2**. The effect of encountering radiomics in the unsure group (likely benign and likely malignant) classified by the expert radiologists.

## Data Availability

The datasets used and/or analysed during the current study are available from the corresponding author on reasonable request.

## Abbreviations

AUC: Area under the curve
CE: Contrast-enhanced
CI: Confidence interval
CT: Computed tomography
FDG: Fluorodeoxyglucose
FSM: Feature selection method
LASSO: Least absolute shrinkage and selection operator
LDA: Linear discriminant analysis
LN: Lymph node
NSCLC: Non-small cell lung cancer
PET: Positron emission tomography
PLS: Partial least squares
SUVmax: Maximum standardised uptake value
