# Supplementary material for: Inferring FDG-PET-positivity of lymph node metastases in proven lung cancer from contrast-enhanced CT using radiomics and machine learning
Source: Eur Radiol Exp. 2022 Sep 15;6:44. doi: 10.1186/s41747-022-00296-8 (PMC9474782; doi:10.1186/s41747-022-00296-8)
Supplement: Supplementary file 1 — Additional file 1: Supplementary Figure 1. Representative lymph node metastases. Supplementary Figure 2. Training and cross-validation with LASSO. Supplementary Figure 3. Performance comparison of radiomics in the unsure group (likely benign and likely malignant) to expert radiologists and the effect of encountering the prediction model. Supplementary Figure 4. Performance comparison of radiomics in the unsure group (likely benign and likely malignant) to expert radiologists plotting the 95% confidence interval AUC. Supplementary Table 1. Performance comparison of radiomics and the two expert radiologists. Supplementary Table 2. The effect of encountering radiomics in the unsure group (likely benign and likely malignant) classified by the expert radiologists. [file 41747_2022_296_MOESM1_ESM.pdf]

# ELECTRONIC SUPPLEMENTARY MATERIAL

## Inferring FDG-PET-positivity of lymph node metastases in proven lung cancer from contrast-enhanced CT using radiomics and machine learning

**Supplementary Figure 1** Representative lymph node metastases

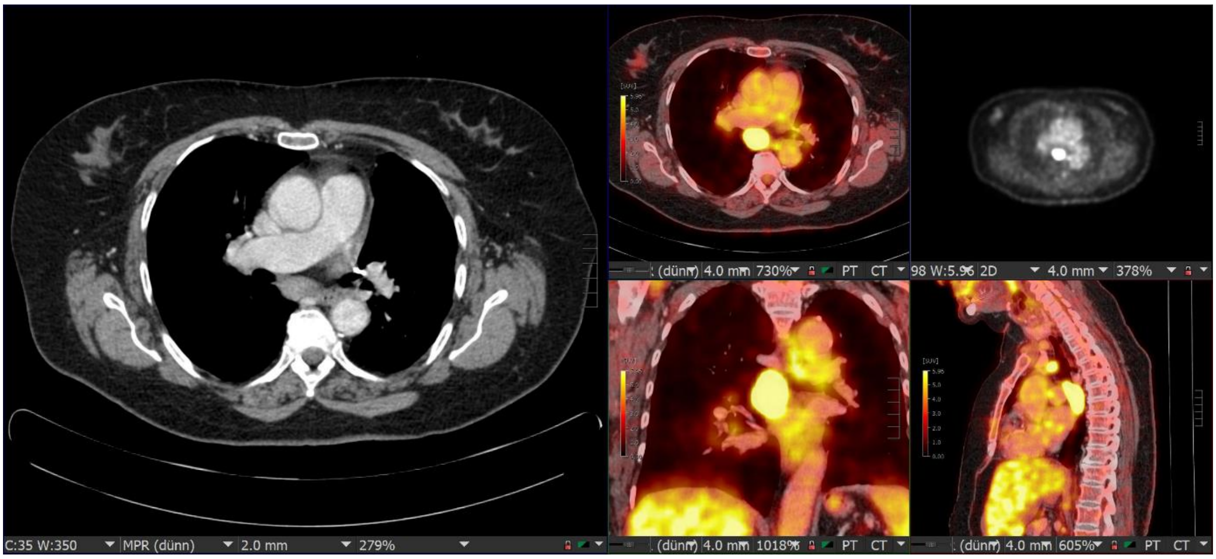

**Supplementary Figure 2** Training and cross-validation with LASSO

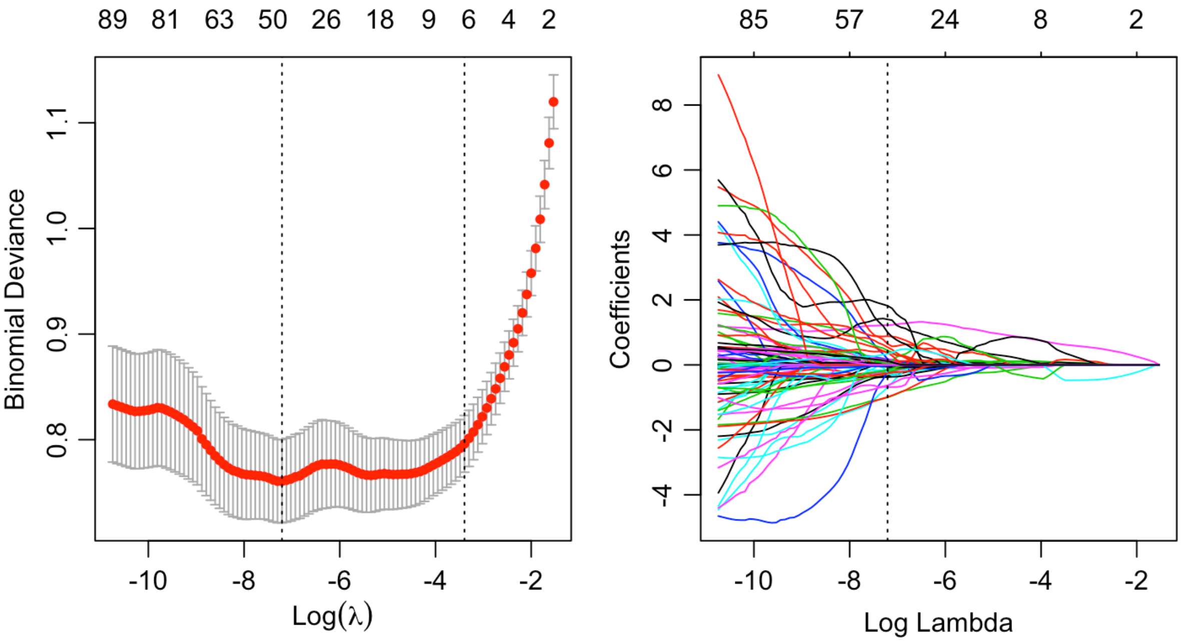


*LASSO* Least absolute shrinkage and selection operator

**Supplementary Figure 3** Performance comparison of radiomics in the unsure group (likely benign and likely malignant) to expert radiologists and the effect of encountering the prediction model

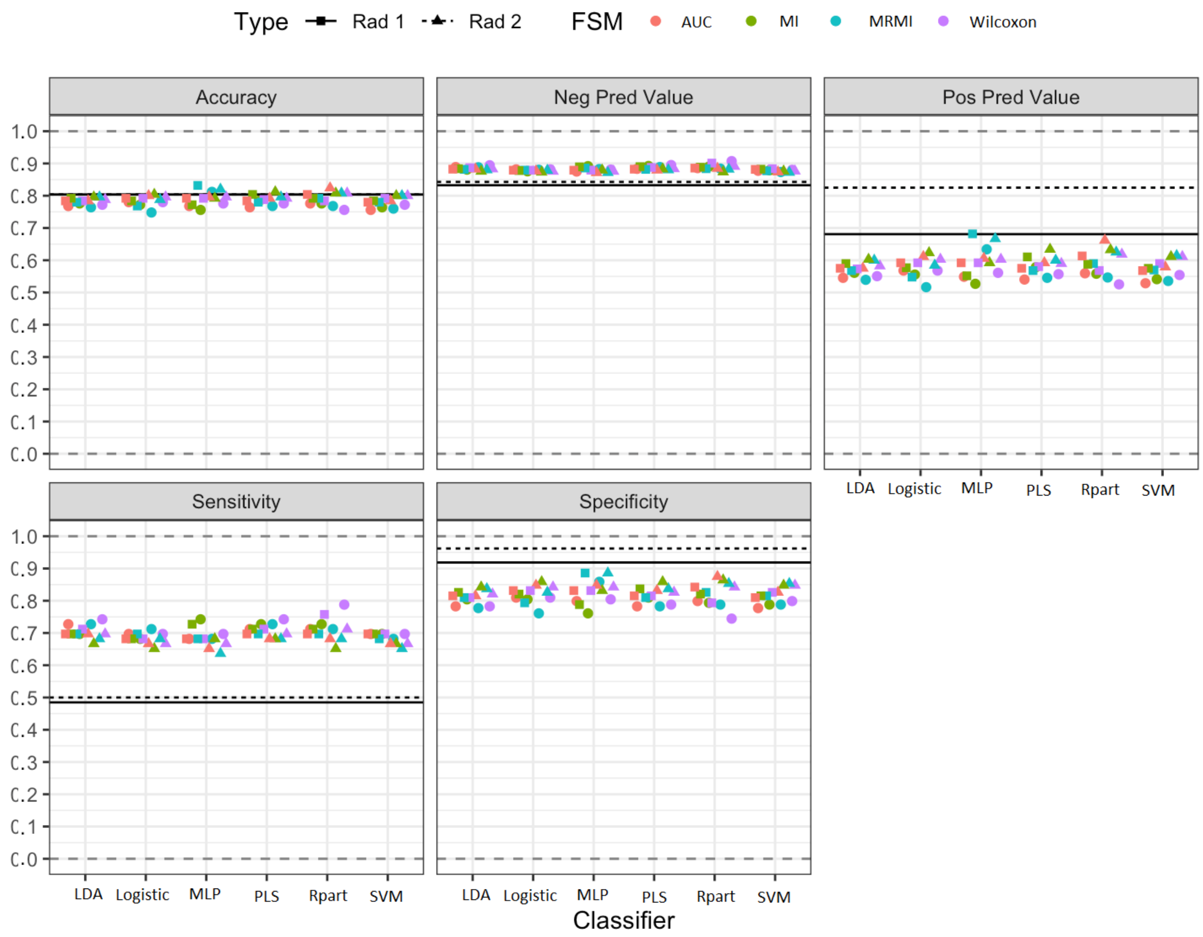


The circles express the prediction of the respective modeling method and analysis, whereas the triangles and squares express the prediction model encountering radiomics in the unsure group (likely benign and likely malignant) classified by both radiologists.

*AUC* Area under the curve, *FSM* Feature selection method, *LDA* Linear discriminant analysis, *Logistic* Logistic regression, *MI* Mutual information, *MLP* multilayer perceptron (neuronal network), *MRMI* Maximum relevance minimum redundancy, NPV Negative predictive value, *PLS* Partial least squares, PPV Positive predictive value, *Rpart* Recursive partition, *SVM* Support vector machine

**Supplementary Figure 4** Performance comparison of radiomics in the unsure group (likely benign and likely malignant) to expert radiologists plotting the 95% confidence interval

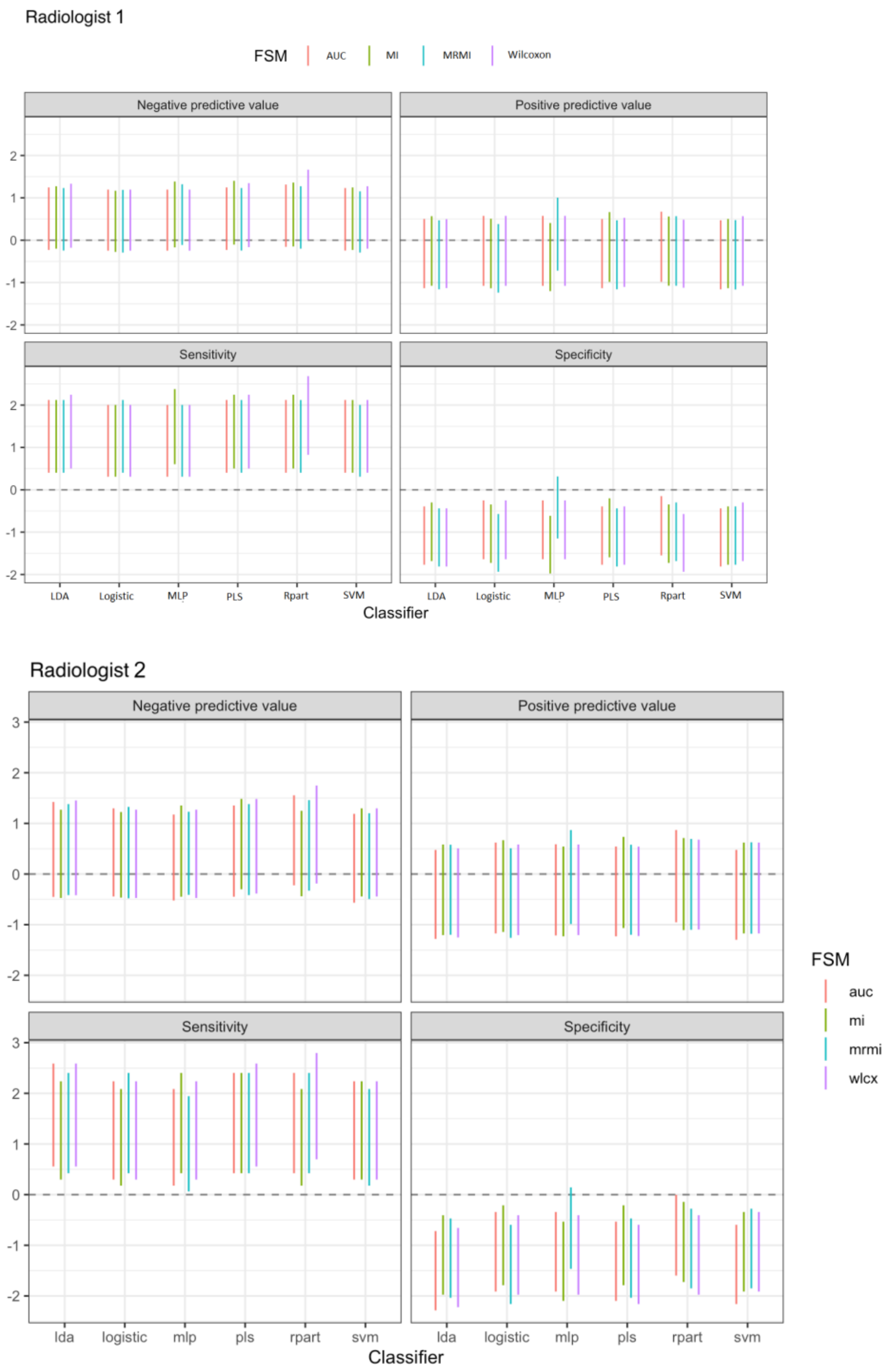


*AUC* Area under the curve, *FSM* Feature selection method, *LDA* Linear discriminant analysis, *Logistic* Logistic regression, *MI* Mutual information, *MLP* multilayer perceptron (neuronal network), *MRMI* Maximum relevance minimum redundancy, NPV Negative predictive value, *PLS* Partial least squares, PPV Positive predictive value, *Rpart* Recursive partition, *SVM* Support vector machine

**Supplementary Table 1** Performance comparison of radiomics and the two expert radiologists

| Classifier | FSM | Accuracy | Sensitivity | Specificity | PPV | NPV |
|---|---|---|---|---|---|---|
| Rad 1 | | 0.8 | 0.48 | 0.92 | 0.68 | 0.83 |
| Rad 2 | | 0.8 | 0.50 | 0.96 | 0.83 | 0.84 |
| LDA | Wilcoxon | 0.77 | 0.74 | 0.78 | 0.55 | 0.89 |
| LDA | AUC | 0.77 | 0.73 | 0.78 | 0.55 | 0.89 |
| LDA | MI | 0.78 | 0.70 | 0.80 | 0.56 | 0.88 |
| LDA | MRMI | 0.76 | 0.73 | 0.78 | 0.54 | 0.89 |
| Logistic regression | Wilcoxon | 0.78 | 0.70 | 0.81 | 0.57 | 0.88 |
| Logistic regression | AUC | 0.78 | 0.70 | 0.81 | 0.57 | 0.88 |
| Logistic regression | MI | 0.77 | 0.68 | 0.80 | 0.56 | 0.88 |
| Logistic regression | MRMI | 0.75 | 0.71 | 0.76 | 0.52 | 0.88 |
| PLS | Wilcoxon | 0.78 | 0.74 | 0.79 | 0.56 | 0.90 |
| PLS | AUC | 0.76 | 0.71 | 0.78 | 0.54 | 0.88 |
| PLS | MI | 0.79 | 0.73 | 0.81 | 0.58 | 0.89 |
| PLS | MRMI | 0.77 | 0.73 | 0.78 | 0.55 | 0.89 |
| SVM | Wilcoxon | 0.77 | 0.70 | 0.80 | 0.55 | 0.88 |
| SVM | AUC | 0.76 | 0.70 | 0.78 | 0.53 | 0.88 |
| SVM | MI | 0.76 | 0.70 | 0.79 | 0.54 | 0.88 |
| SVM | MRMI | 0.76 | 0.68 | 0.79 | 0.54 | 0.87 |
| MLP | Wilcoxon | 0.78 | 0.70 | 0.80 | 0.56 | 0.88 |
| MLP | AUC | 0.77 | 0.68 | 0.80 | 0.55 | 0.88 |
| MLP | MI | 0.76 | 0.74 | 0.76 | 0.53 | 0.89 |
| MLP | MRMI | 0.81 | 0.68 | 0.86 | 0.63 | 0.88 |
| Rpart | Wilcoxon | 0.76 | 0.79 | 0.74 | 0.53 | 0.91 |
| Rpart | AUC | 0.78 | 0.71 | 0.80 | 0.56 | 0.89 |
| Rpart | MI | 0.78 | 0.73 | 0.79 | 0.56 | 0.89 |
| Rpart | MRMI | 0.77 | 0.71 | 0.79 | 0.55 | 0.88 |

*AUC* Area under curve at receiver operating characteristics analysis, *FSM* Feature selection method, *LDA* Linear discriminant analysis, *MI* Mutual information, *MLP* Multilayer perceptron (neuronal network), *MRMI* Maximum relevance minimum redundancy. *NPV* Negative predictive value, *PLS* Partial least squares, *PPV* Positive predictive value, *Rpart* Recursive partition, *SVM* support vector machine

**Supplementary Table 2** The effect of encountering radiomics in the unsure group (likely benign and likely malignant) classified by the expert radiologists

| Type | Classifier | FSM | Accuracy | Sensitivity | Specificity | PPV | NPV |
|---|---|---|---|---|---|---|---|
| Rad 1 | LDA | Wilcoxon | 0.78 | 0.71 | 0.81 | 0.57 | 0.89 |
| Rad 1 | LDA | AUC | 0.78 | 0.70 | 0.82 | 0.57 | 0.88 |
| Rad 1 | LDA | MI | 0.79 | 0.70 | 0.83 | 0.59 | 0.88 |
| Rad 1 | LDA | MRMI | 0.78 | 0.70 | 0.81 | 0.57 | 0.88 |
| Rad 1 | Logistic regression | Wilcoxon | 0.79 | 0.68 | 0.83 | 0.59 | 0.88 |
| Rad 1 | Logistic regression | AUC | 0.79 | 0.68 | 0.83 | 0.59 | 0.88 |
| Rad 1 | Logistic regression | MI | 0.78 | 0.68 | 0.82 | 0.58 | 0.88 |
| Rad 1 | Logistic regression | MRMI | 0.77 | 0.70 | 0.79 | 0.55 | 0.88 |
| Rad 1 | PLS | Wilcoxon | 0.79 | 0.71 | 0.82 | 0.58 | 0.89 |
| Rad 1 | PLS | AUC | 0.78 | 0.70 | 0.82 | 0.57 | 0.88 |
| Rad 1 | PLS | MI | 0.80 | 0.71 | 0.84 | 0.61 | 0.89 |
| Rad 1 | PLS | MRMI | 0.78 | 0.70 | 0.81 | 0.57 | 0.88 |
| Rad 1 | SVM | Wilcoxon | 0.79 | 0.70 | 0.83 | 0.59 | 0.88 |
| Rad 1 | SVM | AUC | 0.78 | 0.70 | 0.81 | 0.57 | 0.88 |
| Rad 1 | SVM | MI | 0.78 | 0.70 | 0.82 | 0.57 | 0.88 |
| Rad 1 | SVM | MRMI | 0.78 | 0.68 | 0.82 | 0.57 | 0.88 |
| Rad 1 | MLP | Wilcoxon | 0.79 | 0.68 | 0.83 | 0.59 | 0.88 |
| Rad 1 | MLP | AUC | 0.79 | 0.68 | 0.83 | 0.59 | 0.88 |
| Rad 1 | MLP | MI | 0.77 | 0.73 | 0.79 | 0.55 | 0.89 |
| Rad 1 | MLP | MRMI | 0.83 | 0.68 | 0.89 | 0.68 | 0.89 |
| Rad 1 | Rpart | Wilcoxon | 0.78 | 0.76 | 0.79 | 0.57 | 0.90 |
| Rad 1 | Rpart | AUC | 0.80 | 0.70 | 0.84 | 0.61 | 0.89 |
| Rad 1 | Rpart | MI | 0.79 | 0.71 | 0.82 | 0.59 | 0.89 |
| Rad 1 | Rpart | MRMI | 0.79 | 0.70 | 0.83 | 0.59 | 0.88 |
| Rad 2 | LDA | Wilcoxon | 0.79 | 0.70 | 0.82 | 0.58 | 0.88 |
| Rad 2 | LDA | AUC | 0.78 | 0.70 | 0.82 | 0.57 | 0.88 |
| Rad 2 | LDA | MI | 0.80 | 0.67 | 0.84 | 0.60 | 0.88 |
| Rad 2 | LDA | MRMI | 0.80 | 0.68 | 0.84 | 0.60 | 0.88 |
| Rad 2 | Logistic regression | Wilcoxon | 0.80 | 0.67 | 0.84 | 0.60 | 0.88 |
| Rad 2 | Logistic regression | AUC | 0.80 | 0.67 | 0.85 | 0.61 | 0.88 |
| Rad 2 | Logistic regression | MI | 0.80 | 0.65 | 0.86 | 0.62 | 0.87 |
| Rad 2 | Logistic regression | MRMI | 0.79 | 0.68 | 0.83 | 0.58 | 0.88 |
| Rad 2 | PLS | Wilcoxon | 0.79 | 0.70 | 0.83 | 0.59 | 0.88 |
| Rad 2 | PLS | AUC | 0.79 | 0.68 | 0.83 | 0.59 | 0.88 |
| Rad 2 | PLS | MI | 0.81 | 0.68 | 0.86 | 0.63 | 0.88 |
| Rad 2 | PLS | MRMI | 0.80 | 0.68 | 0.84 | 0.60 | 0.88 |
| Rad 2 | SVM | Wilcoxon | 0.80 | 0.67 | 0.85 | 0.61 | 0.88 |
| Rad 2 | SVM | AUC | 0.78 | 0.67 | 0.83 | 0.58 | 0.87 |
| Rad 2 | SVM | MI | 0.80 | 0.67 | 0.85 | 0.61 | 0.88 |
| Rad 2 | SVM | MRMI | 0.80 | 0.65 | 0.85 | 0.61 | 0.87 |
| Rad 2 | MLP | Wilcoxon | 0.80 | 0.67 | 0.84 | 0.60 | 0.88 |
| Rad 2 | MLP | AUC | 0.80 | 0.65 | 0.85 | 0.61 | 0.87 |

| | | | | | | | |
|---|---|---|---|---|---|---|---|
| Rad 2 | MLP | MI | 0.79 | 0.68 | 0.83 | 0.59 | 0.88 |
| Rad 2 | MLP | MRMI | 0.82 | 0.64 | 0.89 | 0.67 | 0.87 |
| Rad 2 | Rpart | Wilcoxon | 0.81 | 0.71 | 0.84 | 0.62 | 0.89 |
| Rad 2 | Rpart | AUC | 0.82 | 0.68 | 0.88 | 0.66 | 0.88 |
| Rad 2 | Rpart | MI | 0.81 | 0.65 | 0.86 | 0.63 | 0.87 |
| Rad 2 | Rpart | MRMI | 0.81 | 0.68 | 0.85 | 0.62 | 0.88 |

*AUC* Area under curve at receiver operating characteristics analysis, *FSM* Feature selection method, *LDA* Linear discriminant analysis, *MI* Mutual information, *MLP* Multilayer perceptron (neuronal network), *MRMI* Maximum relevance minimum redundancy. *NPV* Negative predictive value, *PLS* Partial least squares, *PPV* Positive predictive value, *Rpart* Recursive partition, *SVM* support vector machine
